# PAMAM-G4 protect the *N*-(2-hydroxyphenyl)-2-propylpentanamide (HO-AAVPA) and maintain its antiproliferative effects on MCF-7

**DOI:** 10.1038/s41598-023-30144-7

**Published:** 2023-02-28

**Authors:** Alma Alicia Ortiz-Morales, Juan Benjamín García-Vázquez, Manuel Jonathan Fragoso-Vázquez, Martha Cecilia Rosales-Hernández, Leticia Guadalupe Fragoso-Morales, Alan Rubén Estrada-Pérez, José Correa-Basurto

**Affiliations:** 1 Laboratorio de Diseño y Desarrollo de Nuevos Fármacos e Innovación Biotecnológica (Laboratory for the Design and Development of New Drugs and Biotechnological Innovation), SEPI-Escuela Superior de Medicina, Instituto Politécnico Nacional, Plan de San Luis y Díaz Mirón, 11340 Mexico City, Mexico; 2 Departamento de Química Orgánica, Escuela Nacional de Ciencias, Biológicas, Instituto Politécnico Nacional, Prolongación de Carpio y Plan de Ayala, Col. Casco de Santo Thomas, Delegación Miguel Hidalgo, 11340 Mexico City, Mexico

**Keywords:** Cancer, Computational biology and bioinformatics, Drug discovery

## Abstract

Our work group designed and synthesized a promissory compound *N*-(2-hydroxyphenyl)-2-propylpentanamide (HO-AAVPA). The HO-AAVPA is a HDAC1 inhibitor and antiproliferative in cancer cell lines. However, HO-AAVPA is poor water solubility and enzymatically metabolized. In this work, the fourth-generation poly(amidoamine) dendrimer (PAMAM-G4) was used as a drug deliver carrier of HO-AAVPA. Moreover, HO-AAVPA and HO-AAVPA-PAMAM complex were submitted to forced degradation studies (heat, acid, base, oxidation and sunlight). Also, the HO-AAVPA-PAMAM-G4 complex was assayed as antiproliferative in a breast cancer cell line (MCF-7). The HO-AAVPA-PAMAM-G4 complex was obtained by docking and experimentally using three pH conditions: acid (pH = 3.0), neutral (pH = 7.0) and basic (pH = 9.0) showing that PAMAM-G4 captureand protect the HO-AAVPA from forced degradation, it is due to sunlight yielded a by-product from HO-AAVPA. In addition, the PAMAM-G4 favored the HO-AAVPA water solubility under basic and neutral pH conditions with significant difference (F_(2,18)_ = 259.9, *p* < 0.001) between the slopes of the three conditions being the basic condition which solubilizes the greatest amount of HO-AAVPA. Finally, the HO-AAVPA-PAMAM-G4 complex showed better antiproliferative effects on MCF-7 (IC_50_ = 75.3 μM) than HO-AAVPA (IC_50_ = 192 μM). These results evidence that PAMAM-G4 complex improve the biological effects of HO-AAVPA.

## Introduction

**Figure 1:**
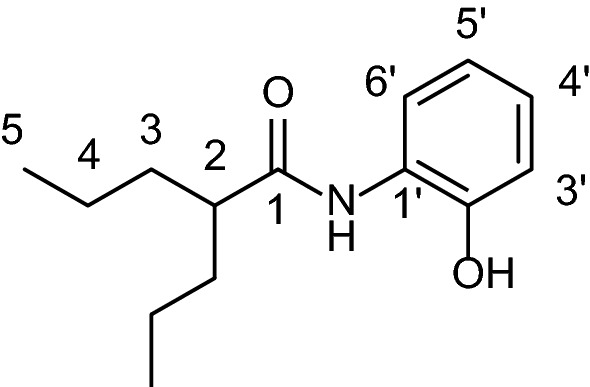
Structure of *N*-(2-hydroxyphenyl)-2-propylpentanamide (HO-AAVPA).

Epigenetics changes can modulate gene expression. These epigenetic changes include protein acetylation and deoxyribonucleic acid (DNA) methylation, among others, without genetic mutations or translocations^1^. The DNA in the cell nucleus is wrapped around proteins called histones to form the nucleosome^2^. The acetylation of Lys residues from histones allows DNA to interact with transcription factor, modulating the protein expression, which is related to several diseases^3^. Histone acetyltransferases (HATs) acetylate Lys residues of histone or nonhistone proteins^4^, whereas histone deacetylases (HDACs) remove the acetyl group^3^. Then, HDAC inhibitors (HDACi) affect the biological functions of different cells from immune system and cancer^5^. Most HDACi are coordinated with a zinc ion (Zn^2+^) in the HDAC active site (Zn^2+^-dependent), causing cell cycle arrest and the differentiation and apoptosis of cancer cells^6^. Valproic acid (VPA) is a HDACi and antiproliferative in cancer cells^7,8^, however, it has certain pharmacological disadvantages due its carcinogenic effects^9^, teratogenic effects^10^, and other side effects^11^. For example, CYP2C9 metabolizes VPA through a dehydrogenation reaction at the aliphatic chain, yielding a reactive metabolite, 2-*n*-propyl-4-pentenoic acid (4nVPA)^12^. In our work group, a set of aryl VPA derivatives was designed containing SAHA (suberoylanilide hydroxamic acid) and VPA scaffolds with the aim of improving the HDAC inhibitory effects and antiproliferative properties of VPA^13^. From this work, we obtained a compound *N*-(2-hydroxyphenyl)-2-propylpentanamide named HO-AAVPA (Fig. 1) (Mexican patent: MX 363,005 B). HO-AAVPA showed antiproliferative effects on cancer cell lines (HeLa, rhabdomyosarcoma, MCF-7, MDA-MB-231 and SKBr3)^13^. Some toxicity studies in a rat model show that HO-AAVPA does not cause liver damage in acute and subchronic treatment, it does not affect organogenesis, and its lethal dose 50 (LD_50_)^14^ is inside the drug toxicity limits recommended^15^. However, during the development and validation of analytical method of HO-AAVPA on reversed-phase high-performance liquid chromatography (RP-HPLC) there was identified a metabolite from a metabolism study into rat liver microsomes^16^ which yield two metabolites^17^. In addition, HO-AAVPA has rapid clearance in pharmacokinetic studies in a rat model^18^. Furthermore, there have not been chemical stability (forced degradation) studies of HO-AAVPA, which are required for all new drugs, to predict the by-products^19^. The chemical structure of HO-AAVPA (amide and phenol groups) can suffer free radical effects, yielding metabolites that could induce side effects as has been reported for other similar compounds^20^. Macromolecules have emerged as promising a drug deliver carriers for administering anticancer drugs due to their greater effect of enhancing permeability and molecular retention^21,22^. The drug deliver carriers based on lipids and polymers are capable to sequester drugs, protect and deliver in some tissues according to pH, temperature, high blood flow, which are useful for chemotherapeutic drugs in cancer^23^. Polyamidoamine dendrimers (PAMAMs) have been studied as drug deliver carriers due to their physicochemical properties able to form drug-dendrimer complexes^24^. The drug-PAMAM complexes improve the pharmacokinetic and pharmacodynamic properties of the drugs because PAMAM protect drugs (favor chemical stability) from physiological environments, increase the water solubility and bioavailability^25^. PAMAM dendrimer has positive protonate amines which could favor the drugs deliver into biological negative environments like cancer^25^.

In this work, molecular docking and experimental assays were performed to form a HO-AAVPA-PAMAM complex. Then, the HO-AAVPA-PAMAM-G4 complex and HO-AAVPA were submitted to forced degradation studies. Afterward, the chemical characterization of the HO-AAVPA-PAMAM-G4 complex was performed to be evaluated as antiproliferative on a breast cancer cell line (MCF-7).

## Material and methods

The HO-AAVPA was synthesized in our laboratory^13^. The PAMAM-G4 was purchase from Sigma-Aldrich, México (412,449-10G). The forced degradation stress studies of HO-AAVPA and the synthesis of the HO-AAVPA-PAMAM complex were monitored by liquid chromatographic using a 1260 Infinity series HPLC system (Agilent Technologies) equipped with a quaternary pump (G1311B), autosampler (G1316A), column oven (G1316A), detector by diode array (G1315C) and OpenLab CDS EZChrom software version A.04.08 to analyze the results. The infrared spectroscopy (IR) characterization was carried out with a Perkin Elmer model Spectrum 2000. The ^1^H and ^13^C NMR characterization was carried out with a Bruker ASCEND™ 750 MHz. The mass spectrometry (MS) characterization was carried out with a 1290 Infinity II series UHPLC system coupled through an electrospray ionization (ESI) source with Jet Strem technology to a 6545Q-TOF/MS system (Agilent Technologies), and MassHunter software LC/MS Data acquisition version B.06.01 was used for data acquisition. MassHunter LC/MS Quantitative Analysis version B.07.00 was used for data analysis.

### Chemical synthesis of the HO-AAVPA-PAMAM-G4 complexes

The chemical synthesis of the HO-AAVPA-PAMAM-G4 complex was carried out under different pH conditions. The PAMAM-G4 was dried using high purity nitrogen gas. Subsequently, three stock solutions of PAMAM-G4 were prepared with deionized water (Milli-Q) at pH 7.0 (adjusted with 1 M NaOH), pH 3.0 (adjusted with 1 M HCl) and pH 9.0 (adjusted with 1 M NaOH) at 10 mg/mL. The HO-AAVPA-PAMAM-G4 complexes were synthesized by adding HO-AAVPA (0.5 mg) to different PAMAM-G4 concentrations (10–50 μM) prepared with 500 μL of deionized water at pH 7.0, pH 3.0 and pH 9.0. The solutions were vortexed for 2 min and then shaken for 24 h at 25 °C. Finally, the samples were centrifuged at 5000 rpm for 10 min using the supernatant to measure the HO-AAVPA concentrations by HPLC.

### Chemical characterization of the HO-AAVPA-PAMAM-G4 complex

The HO-AAVPA-PAMAM-G4 complex was dried under a vacuum to carry out its chemical characterization:

### HPLC

Samples of HO-AAVPA, PAMAM and HO-AAVPA-PAMAM-G4 complex were used to compare the HO-AAVPA concentrations before and after the synthesis of the HO-AAVPA-PAMAM-G4 complexes. The chromatographic conditions employed a reverse-phase Zorbax SB-C18 column at 35 °C. The flow rate was 1.0 mL/min, injecting 10 µL. The gradient consisted of mobile phase A (Acetonitrile) and mobile phase B (Water) during 13 min. The gradient started at 60%A, increasing to 80%A in 6 min, then held until 8 min. Starting conditions were returned by 10 min, and a 3 min re-equilibration time was included mobile phase included isocratic elution of a mixture of water 60% and acetonitrile 40%. The maximum absorbance wavelength was 242 nm. The analytical method used for the quantification of the HO-AAVPA was developed and validated.

### FT infrared spectrometry

HO-AAVPA-PAMAM-G4 complex (cm^−1^): 3540 (O–H), 2985.5 and 2946.2 (C–H); 1741.5 (C=O); 1474, 1448, 1047.3 (N–C); 1373.9 (C–O–H) and 1241.1 (C–O).

### ^1^H and ^13^C NMR spectra

HO-AAVPA-PAMAM-G4 complex: ^1^H NMR (750 MHz, D_2_O): δ 8.10 (s, 1H, NH), 7.5–6.5 (m, due to HO-AAVPA-Ar-H), 3.29 (s, 1H, H-3), 3.22 (s, 1H, H-4), 3.12 (s, 1H, H-5), 2.81 (s, 1H, H-1), 2.62 (s, 1H, H-6), 2.40 (s, 1H, H-2), 1.5–1.2 (m, due to HO-AAVPA-CH_2_-), 0.7 (s, due to HO-AAVPA-CH_3_-). ^13^C NMR ppm (187.5 MHz, D_2_O) δC: 175.0 (C), 174.5 (C), 174.4 (C), 174.3 (C), 51.2 (CH_2_), 49.0 (CH_2_), 41.1 (CH_2_), 41.0 (CH_2_), 40.9 (CH_2_), 40.3 (CH_2_), 39.9 (CH_2_), 39.7 (CH_2_), 38.6 (CH_2_), 36.7 (CH_2_), 32.7 (CH_2_).

### Mass spectrometry

The exact mass of HO-AAVPA, PAMAM-G4 and HO-AAVPA-PAMAM-G4 complex determined by LC-ESI-QTOF-MS/MS were: HO-AAVPA = 235.1645 g/mol, PAMAM-G4 = 14,214.3425 g/mol, HO-AAVPA-PAMAM-G4 complex = 16,398.1767, 16,882.4909 and 16,948.9560, meaning that 9, 11 and 12 HO-AAVPA molecules were coupled on PAMAM-G4, respectively.

### Force degradation studies of HO-AAVPA and HO-AAVPA-PAMAM complex

To explore the chemical stability of HO-AAVPA and the HO-AAVPA-PAMAM-G4 complex, these systems were submitted to forced degradation studies under different stress conditions: acid, base, oxidation, heat, and sun light. Stock solutions of the HO-AAVPA and HO-AAVPA-PAMAM-G4 complex (2 mg/mL) were prepared. From the stock solution, 2 mL was placed in 10 mL flasks to be treated under the following conditions:

- *Acid-basic conditions* For the acid condition, 1 mL of 0.1 M HCl was added to stock solution. For the basic condition, 1 mL of 0.1 M NaOH was added to stock solution to be maintained for 24 h. Then, the reactions were neutralized using 1 mL of 0.1 M NaOH for acidic and 1 mL of 0.1 M HCl for basic conditions. Finally, each sample was mixed with the mobile phase (ACN: Water 60:40) diluted at 0.4 mg/mL.
- *Oxidative* 1 mL of 3.0% H_2_O_2_ was added to stock solution to be maintained for 8 h and was then brought to volume with the mobile phase.
- *Heat* The stock solution was mixed with mobile phase in a closed-bottle which was subjected at 50 °C for 6 h in a heating oven.
- *Sunlight* The stock solution was mixed with mobile phase and exposed to sunlight for 24 h.

### Water solubility test

The water solubility assays of the HO-AAVPA (0.5 mg) was carried out using increased concentrations of PAMAM-G4 (10–50 μM). To measure the free HO-AAVPA (intrinsic solubility), 0.5 mg of the HO-AAVPA was dissolved in 500 μL of deionized water at pH 7.0, pH 3.0 and pH 9.0. To quantify the concentration of HO-AAVPA-PAMAM-G4 complexes, calibration curves were prepared from 4.0 to 1200 μg/mL using a stock solution of 2 mg/mL of the HO-AAVPA in ethanol. The calibration curves were prepared for each pH condition mentioned. The concentration of HO-AAVPA present in the HO-AAVPA-PAMAM-G4 complex at each pH (acid, basic and neutral) was determined by the equation of the line obtained from the corresponding calibration curve. The water solubility of HO-AAVPA at different PAMAM-G4 concentrations were evaluated by HPLC following the procedures reported elsewhere^26,27^.

### Measuring the dissociation constant (*Kd*)

#### *Kd* in solid medium

2 mg of the HO-AAVPA was placed in different concentrations of PAMAM-G4 (0.0 to 10 mg/mL) in Eppendorf tubes and adjusted with 500 μL of water at pH 7.0. To determine the PAMAM saturation with HO-AAVPA, the samples were shaken for 24 h in the dark at 25 °C. Then, the Eppendorf tubes were centrifuged at 5000 rpm for 5 min. The water solubility profiles were obtained by plotting the concentration of the HO-AAVPA against different PAMAM-G4 concentrations. The stability constant was determined from the solubility profiles using the Higuchi and Connors equation (Eq. 1) for multiple binding sites^26^.

1 $$ {\varvec{S}}_{{\varvec{t}}} = {\varvec{S}}_{{\varvec{o}}} + \frac{{{\varvec{nK}}_{{{\varvec{n}}:1}} \left( {{\varvec{S}}_{{\varvec{o}}} } \right)^{{\varvec{n}}} }}{{1 + {\varvec{K}}_{{{\varvec{n}}:1}} \left( {{\varvec{S}}_{{\varvec{o}}} } \right)^{{\varvec{n}}} }}{\varvec{L}}_{{\varvec{t}}} $$

where S_t_ is the observed molar solubility of the compound, K_n:1_ is the average equilibrium stability constant per binding site for a n:1 complex, n is the number of HO-AAVPA molecules per each PAMAM-G4 molecule that form the HO-AAVPA-PAMAM-G4 complex, Therefore, *S*_*o*_ is the molar solubility intrinsic of the HO-AAVPA and L_t_ is the total molar concentration of the PAMAM-G4.

#### *Kd* in liquid medium

The *Kd* was evaluated at pH 7.0 using a stock solution (2 mg/mL) of the HO-AAVPA. From stock aliquots, different concentrations of HO-AAVPA were placed in Eppendorf tubes containing PAMAM-G4 at 50 μM. The final volume was adjusted with water at pH 7.0–1 mL. Then, the solutions were vortexed for 2 min and finally shaken for 24 h at 25 °C. These samples were centrifuged at 5000 rpm for 10 min, and the supernatant was recovered to measure the HO-AAVPA by HPLC.

From the supernatant, 500 μL was transferred to centrifuge filtration tubes (Amicon^®^ Ultra-0.5 L Centrifugal Filters, Ultracel^®^-3 K, Brand: Millipore) for ultracentrifugation at 5000 rpm in 10 min intervals until a steady state was obtained (determined by the maintenance of the unfiltered volume). Finally, the unfiltered phases were recovered and placed in 2 mL vials and adjusted to a volume of 500 μL with water pH 7.0; in the same way, they were filtered and analyzed by HPLC to calculate the *Kd*^27,28^. To quantify HO-AAVPA coupled on PAMAM-G4, a calibration curve of the compound (4.0–1200.0 μg/mL) was prepared. The curve was performed in triplicate from a stock solution of 2 mg/mL and analyzed by HPLC.

### HO-AAVPA-PAMAM-G4 complex: In silico

The molecular docking of HO-AAVPA on PAMAM was achieved with the AutoDock 4.2 program^29^. PAMAM-G4 was evaluated in three pH states: acid (amine fully protonated, + charge), basic (unprotonated amine) and neutral (protonated primary amine, + charge). The neutral and basic PAMAM-G4 were obtained from the dendrimer construction toolkit (http://www.physics.iisc.ernet.in/~maiti/dbt/home.html) whereas our work group created the acid PAMAM-G4^24^. Subsequently, polar hydrogen atoms were added, and the partial charges (Kollman) and solvation parameters were added with the AutoDockTools 1.5.6 program. The docking studies were carried out using a blind docking procedure to explore the whole PAMAM-G4 structure under a grid box: 126 Å^3^ and grid spacing: 0.375 Å^3^ using the Lamarckian genetic algorithm, with a maximum number of energy evaluations 1 × 10^7^ and a population of 100 randomized individuals. Molecular docking studies were used to saturate PAMAM-G4 with HO-AAVPA to identify the number of molecules that can be accommodated on PAMAM to form a HO-AAVPA-PAMAM-G4 complex.

### Biological evaluation

MCF-7 (breast cancer cell line) and 3T3-NIH (fibroblasts cell line) cells were used for the antiproliferative assays of HO-AAVPA, PAMAM-G4 and HO-AAVPA-PAMAM-G4 complex. Cells were cultured in DMEM (Dulbecco's Modified Eagle Medium) (Gibco) supplemented with 10% FBS (Fetal Bovine Serum) (Biowest) and 1% Amphotericin B/Penicillin/Streptomycin (Biowest) in a humidified environment at 37 °C and 5% CO_2_; once the cells reached the confluence, they were detached with PBS-EDTA for MCF-7 and trypsin–EDTA [Biowest] for (3T3-NIH), and seed in 96-well plates using 5000 cells/well, and incubated at 37 °C with 5% CO_2_ for 24 h to allow their adherence.

From a HO-AAVPA stock solution (0.85 mM), different concentrations were done 12.5, 25, 50, 100 and 200 µM (in DMEM with DMSO 1%) close to its reported IC_50_ = 192 µM^13^. The PAMAM concentrations employed were into nontoxic 12.5, 25, 50, 75 and 100 µM^30^. Finally, the HO-AAVPA-PAMAM complex stock solution was 1.15 mM (in water) diluting to 12.5, 25, 50, 100 and 200 µM in DMEM with 14% water. All solutions were filtrated through a sterile acrodisc of 0.45 μM PVDF.

### Cell proliferation assay

The antiproliferative effects of HO-AAVPA, PAMAM-G4 and HO-AAVPA-PAMAM-G4 complex on cells were measured using the MTT (3-(4,5-dimethylthiazol-2-yl)-2,5-diphenyltetrazolium bromide) assay. After 48 h of treatment of cells, the medium from each well was removed and replaced with 20 μL of MTT, and the cells were incubated for 4 h. Then, the medium was removed and replaced with 100 μL of DMSO. The absorbance of the samples was measured at 550 nm by a SCIENTIFIC MULTISKAN EX reader from Thermo. The experiments were performed in duplicate with n = 8 for each concentration.

### Statistical analysis

The statistical analysis of the data was carried out in the GraphPad Prism 8.0.1 program. The results of the solubility and dialysis tests were analyzed by linear regression (n = 3). The solubility curves were contrasted by comparing their slopes by the F test.

## Results and discussion

**Figure 2:**
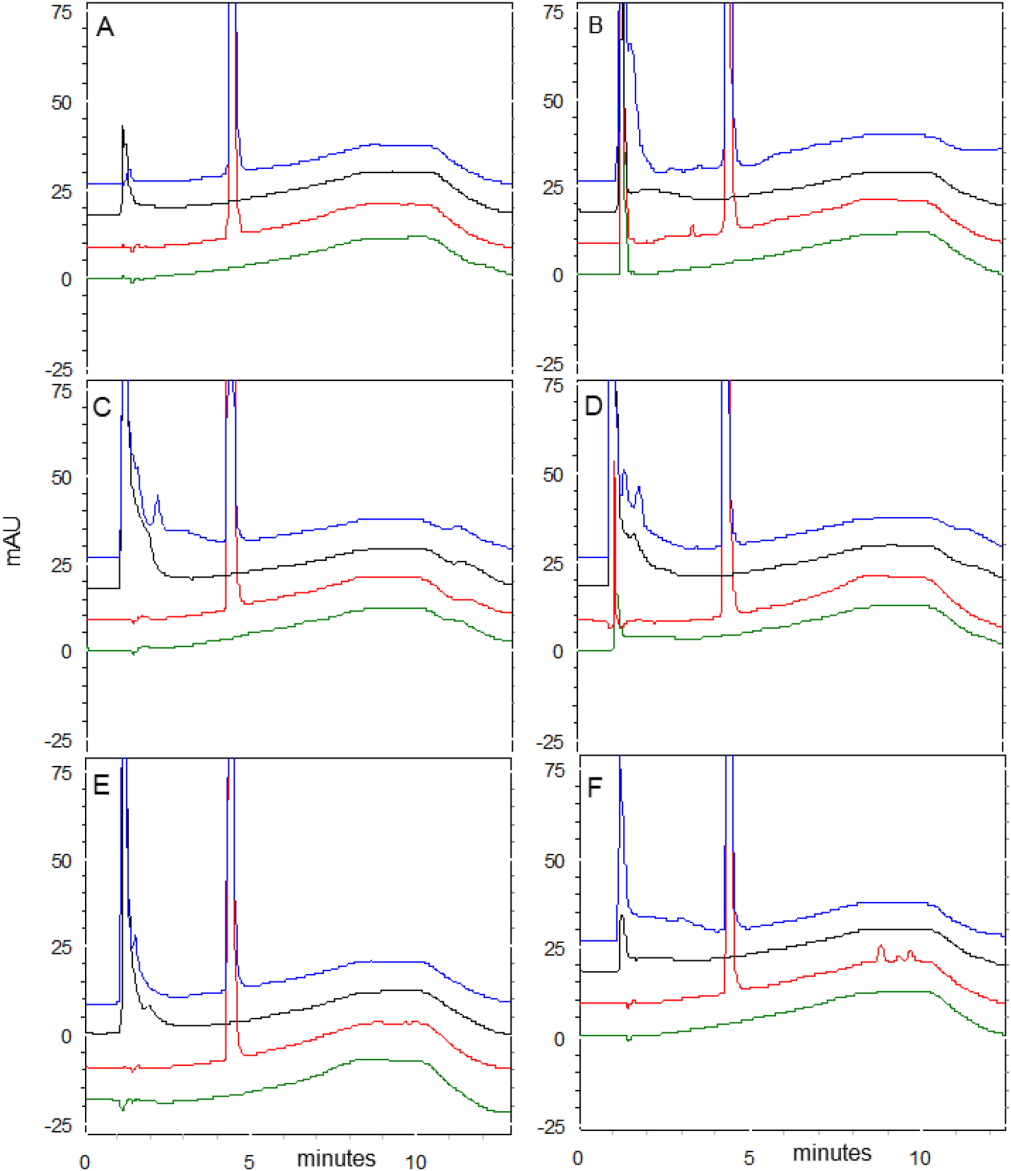
HO-AAVPA and HO-AAVPA-PAMAM complex submitted to forced stress. (**A**). No forced degradation stress, (**B**) H_2_O_2_, (**C**) acid conditions, (**D**) basic conditions, (**E**) heat condition (**F**) sunlight 24 h. A reagent blank was submitted for each condition. Green (mobile phase), red (HO-AAVPA), black (PAMAM) and blue (HO-AAVPA-PAMAM complex).

**Figure 3:**
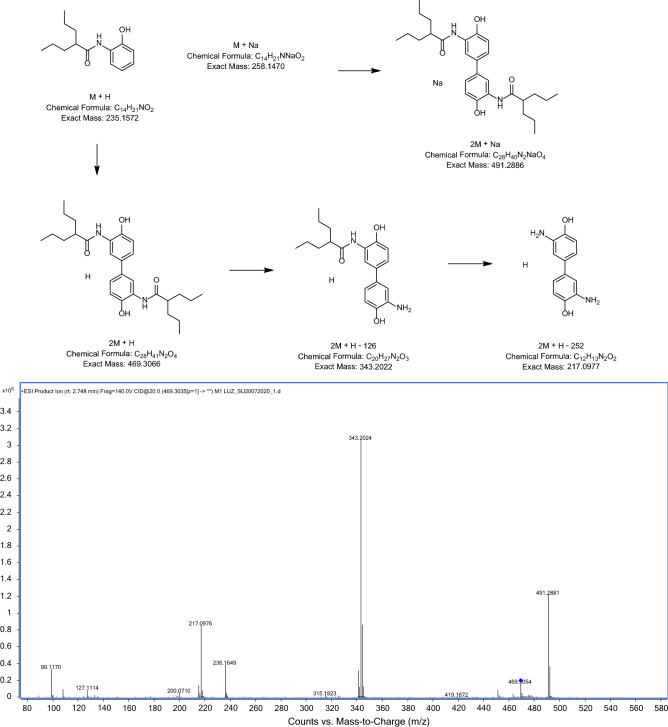
LC–MS/MS fragmentation pattern of HO-AAVPA, identifying some by-products generated during sun exposure.

**Figure 4:**
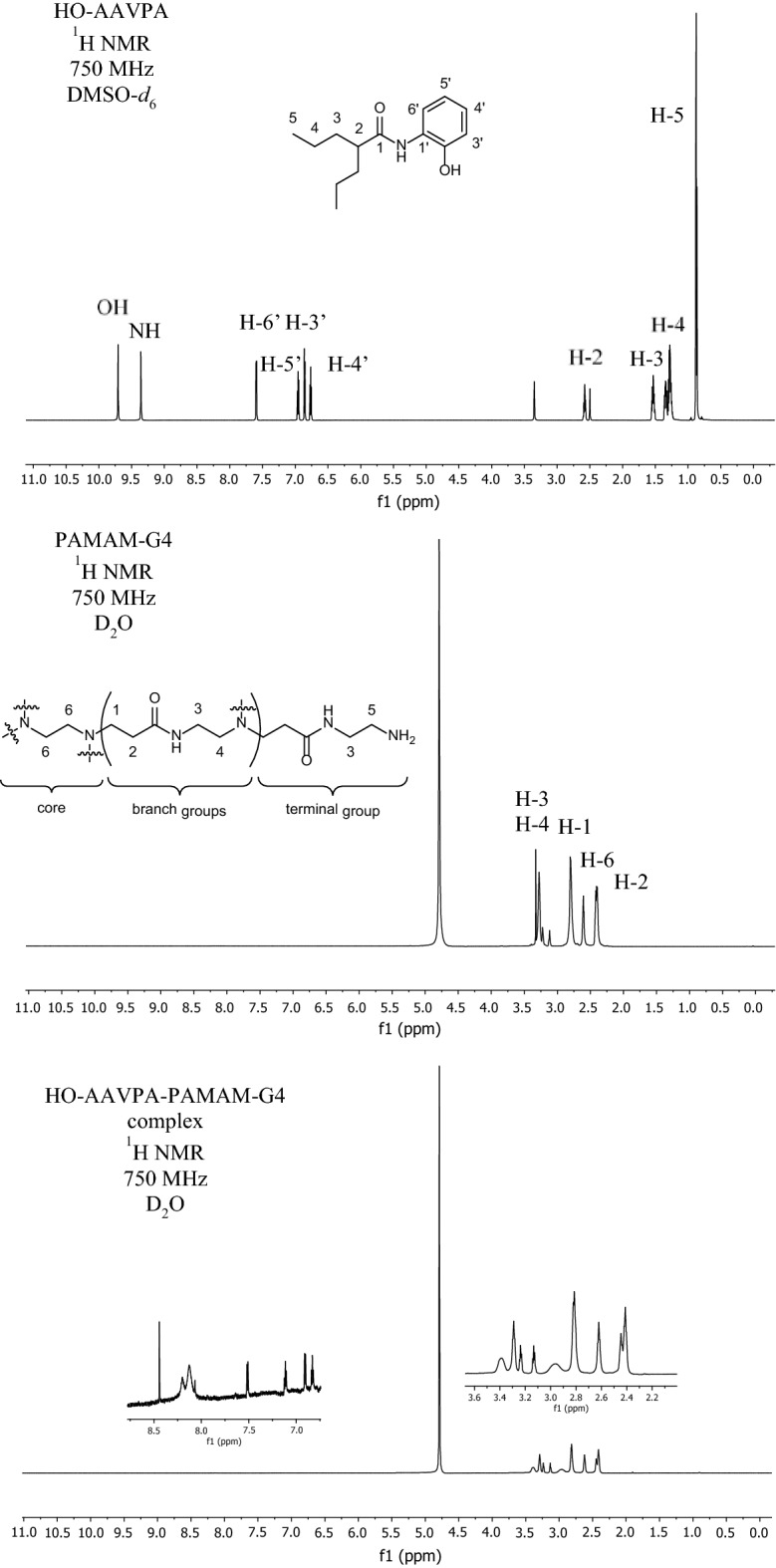
^1^H NMR of HO-AAVPA (upper), PAMAM (medium) and HO-AAVPA-PAMAM complex (lower).

**Figure 5:**
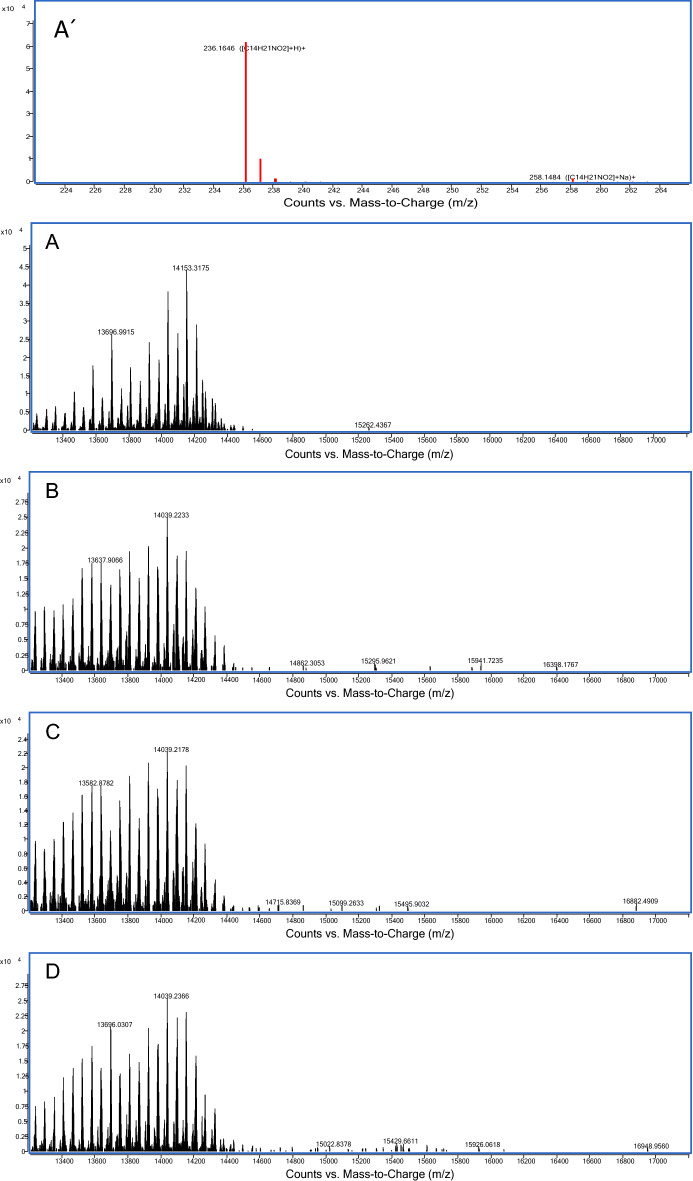
(**A**) Mass spectrum of HO-AAVPA (C_14_H_21_NO_2_ + H)^+^. (**A**) Spectrum obtained by deconvolution of the resolved isotope of the PAMAM. (**B**–**D**) Spectrum obtained by deconvolution of the resolved isotope of the HO-AAVPA-PAMAM-G4 complex.

**Figure 6:**
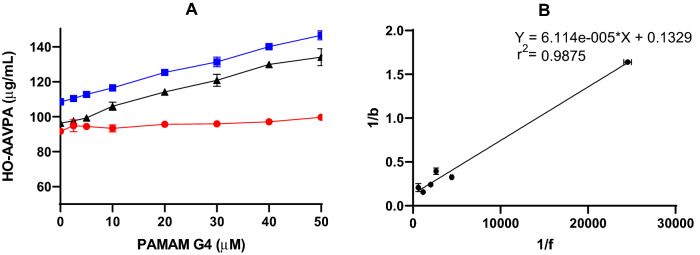
(**A**) Increase of water solubility of HO-AAVPA with increased concentration of PAMAM-G4. Blue (basic), black (neutral) and red (acid). The points represent the mean ± standard error of the mean (SEM). The lines were compared by line regression and the F statistic test (F_(2,18)_ = 259.9, *p* < 0.001). (**B**) Dialysis of HO-AAVPA-PAMAM-G4 complex plotted using the Scatchard-Klotz equation. The dissociation constant = 1556.17 M^−1^. Eleven HO-AAVPA molecules per PAMAM-G4 were counted according to the Scatchard-Klotz equation. Points represent the mean ± SEM for **x** and **y**.

**Figure 7:**
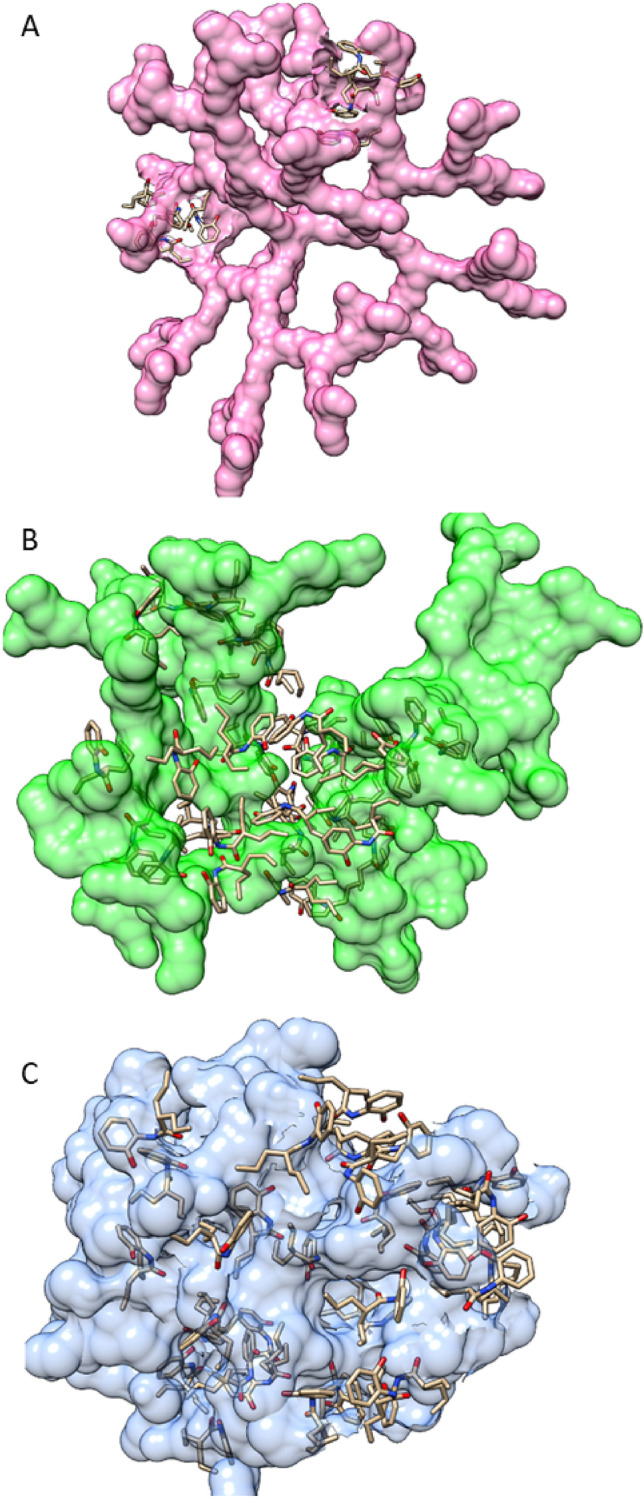
HO-AAVPA docked on PAMAM-G4: (**A**) acid, (**B**) neutral and (**C**) basic conditions. The 3D structures were drawn with PyMol program^54^.

**Figure 8:**
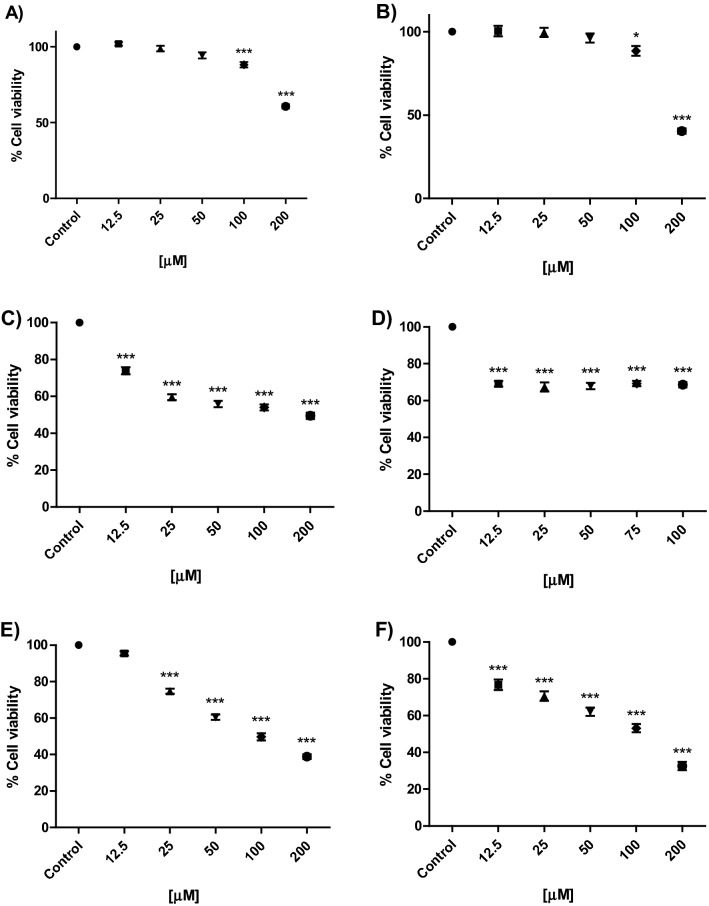
Cell viability of 3T3 NIH fibroblasts and MCF-7 breast cancer cells. Antiproliferative assays of (**A**) HO-AAVPA, (**C**) PAMAM-G4 and (**E**) HO-AAVP-PAMAM-G4 complex on 3T3 NIH fibroblasts and (**B**) HO-AAVPA, (**D**) PAMAM-G4, (**F**) HO-AAVP-PAMAM-G4 complex on MCF-7 breast cancer cells. All treatments were carry out for 48 h with n = 8 duplicate*** < 0.05; treatment versus control.

Drug stability studies are required for all compounds with future human medical uses. Our work group designed and synthesized a set of VPA derivative selecting a compound named HO-AAVPA, it is anti-proliferative in breast cancer cells^13^. However, the HO-AAVPA exposed to rat microsomes generate two metabolites^16^. In addition, HO-AAVPA has low water solubility affecting its pharmacokinetic parameters^18^. Therefore, the compound was mixed with copolymers to improve its water solubility, however, its antiproliferative effects decrease on MDA-MB-231^31^ and with liposomes have antiproliferative effects on fibroblast^32^. In addition, HO-AAVPA has not been subjected to chemical stability studies which are required for all drugs used in humans^19^. Then, the force stress studies are required for HO-AAVPA due to it has an amide and a phenol groups which could suffer chemical changes by environment conditions and induce biological effects^33^. Furthermore, the HO-AAVPA was subjected to a forced stress degradation study as is reported elsewhere^34^ to discard the formation of by-products^35^. The forced stress degradation studies allow to measure the chemical stability of a drug by exposing a compound to different extreme physicochemical conditions, such as acid, alkaline, oxidation, heat, and sunlight^36^. Drugs can be degraded by hydrolysis^37^, oxidative stress^38^. These force degradation studies are needed to determine how to handle the drugs during the synthesis stages as well as in manufacturing stages for human use, including routes of administration, pharmaceutical presentations, etc.^34^. The chemical degradation assays on HO-AAVPA were followed using HPLC (Fig. 2) to separate the generate by-products to be analyzed (molecular weight) by LC–MS/MS as was for ezetemibe^39^. Once the HO-AAVPA was subjected to force degradation studies (acid and alkaline conditions, oxidation, heat, sunlight, the oxidation), only the sunlight generates a by-product that correspond to dimmer HO-AAVPA (Fig. 3). The dimmer formation of HO-AAVPA is possible by sunlight due it generates free radicals^40^ able to form by-products as dimers^41^. Additionally, it is know that HO-AAVPA is a free radical scavengers in cell culture which could be due to its phenol group^42^. These results suggest that HO-AAVPA must be stored in dark containers to protect it from sunlight during the synthesis and handling for preclinical experimental assays and for future human uses. Because HO-AAVPA is metabolize in vitro by rat microsomes^16^ and sunlight can form dimmers (Fig. 3), HO-AAVPA was coupled to PAMAM-G4, reported elsewhere as well ion and drug carrier^43,44^. The HO-AAVPA-PAMAM was built to be subjected to force degradation stress studies with the aim to evaluate the PAMAM protection properties on HO-AAVPA. The results show that PAMAM-G4 is capable to carry out and protect the HO-AAVPA due to there were not identified any by-products (Fig. 2). Previously to the experimental assays, the HO-AAVPA-PAMAM complex was full chemically characterized. The FTIR shows a peak at 3290 cm^−1^ due to the stretching vibration of the –NH– group and the bands that reach a maximum of 1644 and 1560 cm^−1^ corresponding to the amides (–CO– NH–) as reported Zhang et al.^45^ for PAMAM. The FTIR for HO-AAVPA show peaks at: 3259 cm^−1^ (N–H); 2966 and 2929 cm^−1^ (C–H); 1626 cm^−1^ (C=O); 1603 cm^−1^ (C=C aromatic); overtone vibrations of approximately (1640–2100 cm^−1^) and 1390.5 cm^−1^ (C–O–H); as well as absorption at 750 cm^−1^, which indicates the substitution at *ortho* position of the aromatic ring. Finally, the FTIR for HO-AAVPA-PAMAM-G4 complex show peaks at: 3540 cm^−1^ (O–H), which indicates the formation of hydrogen bonds; 2985.5 and 2946.2 cm^−1^ (C–H); 1741.5 cm^−1^ (C=O); and 1474 cm^−1^, 1448, 1047.3 cm^−1^ (N–C); 1373.9 cm^−1^ (C–O–H) and 1241.1 cm^−1^ (C–O). Regarding to ^1^H NMR for the HO-AAVPA-PAMAM-G4 complex, there are original signals affected due to the complex formation corresponding to the hydrogen from the amide group of HO-AAVPA and those from the hydrogens from the tertiary amines of PAMAM which suggest hydrogen bond interactions (Fig. 4). Regarding to the LC–MS/MS results, the HO-AAVPA show a m/z = 235.1556 (Fig. 5A), with an error of 0.4 ppm^46^. Regarding to the HO-AAVPA-PAMAM-G4 complex (Fig. 5B–D), the deconvolution analyses showed different m/z 16,398.1767, 16,882.4909 and 1648.9560, meaning that 9, 11 and 12 HO-AAVPA molecules were coupled on PAMAM-G4, respectively. These signals are not shown on PAMAM-G4^46^ (Fig. 5A). Making this HO-AAVPA-PAMAM-G4 complex was to improves the water solubility of HO-AAVPA due it has low water solubility (high hydrophobicity) which difficulties the in vitro studies and show a decreased bioavailability^18^. Furthermore, the PAMAM-G4 could protect HO-AAVPA from environmental damage (sunlight and cytochromes) and improved the water solubility due to enhancer retention phenomenon^25,47^. There are studies about the favored water solubility of dendrimers, which are dependent on pH and concentration^24^. Furthermore, HO-AAVPA was coupled on PAMAM-G4 experimentally using acid, neutral and alkaline pH conditions. The results showed an increased water solubility of HO-AAVPA in basic and neutral conditions (Fig. 6A) of 96.35–134.11 μg/mL and 108.56–146.60 μg/mL respectively, while in acid medium of 91.71–99.68 μg/mL. The statistical analysis shows a statistically significant difference (F_(2,18)_ = 259.9, *p* < 0.001) between the slopes of the three conditions, the basic condition being the one that solubilizes the greatest amount of HO-AAVPA. In agreement with other reports with methotrexate-PAMAM-G4 in which the PAMAM-PEGylation maximizes the drug loading making it possible to identify multiple binding sites^27^ The HO-AAVPA measure during the HO-AAVPA-PAMAM-G4 complex formation was achieve as is reported elsewhere^48^ getting a validation method and linear curve from 4 to 1200 μg/mL (data not shown). The determination of the dissociation constant (*Kd*) was carried out by two methods. The first, a dialysis method using the Scatchard-Klotz equation^49^ which show a dissociation constant of 1556.17 M^−1^ suggesting eleven HO-AAVPA molecules per each PAMAM-G4 molecule (Fig. 6B). These results are agreement with another study on 5-fluorouracil on PAMAM which increased the drug capture depending of PAMAM-G4 gradually increased^50^. The second method used to determine the *Kd* was obtained applying the Higuchi and Connors equation (Eq. 1), finding a stability constant of 1984.81 M^−1^ and an equilibrium constant of 5.03 × 10^−4^ M of HO-AAVPA on PAMAM-G4. In both cases, HO-AAVPA was sequestered by PAMAM-G4. The stability constant value indicates that the association between HO-AAVPA and PAMAM-G4 is a reversible supramolecular interaction, which is a relevant result for the use of PAMAM-G4 systems in controlled release applications^28^. The results indicate that the water solubility of HO-AAVPA increases using PAMAM-G4 as carrier (Fig. 6A). According to the classification of the phase solubility diagram of Higuchi and Connors (Eq. 1), the HO-AAVPA solubility profiles correspond to the type A_L_ phase diagrams, which support the formation of supramolecular complexes with a unique stoichiometry of type *n:1* between HO-AAVPA and PAMAM-G4. The theoretical docking studies replicate the experimental HO-AAVPA-PAMAM-G4 complex stoichiometry at acid conditions indicating a fast release due to open cavities of repulsive internal positive charges (Fig. 6A). In addition, the PAMAM-G4 was saturate with HO-AAVPA using docking. The results show that PAMAM-G4 accepts 35 HO-AAVPA molecules under basic conditions, 40 under neutral conditions and 11 under acid conditions. The docking results showed better affinity of HO-AAVPA on PAMAM-G4 in basic and in neutral conditions than in acid conditions according to their free energies (ΔG) − 5.94 kcal/mol, − 6.52 kcal/mol and − 4.59 kcal/mol, respectively. In acid medium, there is little interaction between the HO-AAVPA and PAMAM-G4, although it is protonated in all amines which induce repulsion effects due to the positive charges allowing to capture HO-AAVPA, but there not enough retention^51^. These results are agreed with other results in which cro-molyn, methotrexate and fusidic acid on PAMAM-G4 showing higher binding affinity under neutral and basic than acid conditions^24^. The docking studies depicted nonbond interactions (ionic, hydrogen bonds and hydrophobic) which engage the HO-AAVPA into PAMAM-G4 cavities agree with other reports^24^ being principally hydrogen bonds (Fig. 7). Once demonstrated that PAMAM-G4 protects the HO-AAVPA, it was interesting to explore its antiproliferative effects as HO-AAVPA-PAMAM-G4 complex. First, PAMAM was evaluated at different concentrations below to 50 μM due to upper to these concentrations it is cytotoxic on MCF-7^30^ as other reports^52^. In this work HO-AAVPA, PAMAM-G4 and HO-AAVPA-PAMAM-G4 complex were assayed as antiproliferative in the MCF-7 and 3T3 NIH cell lines (Fig. 8). HO-AAVPA and PAMAM-G4 assayed separately showed little antiproliferative responses (Fig. 8). It is due to HO-AAVPA is not dissolved adequately in water solutions contrary to PAMAM. Regarding to PAMAM-G4, it was assayed low 50 μM in which it is not toxic^30^. The results show that the antiproliferative activity of the HO-AAVPA-PAMAM-G4 complex on the MCF-7 cell line showed an IC_50_ = 75.3 μΜ (Fig. 8), which is lower than the IC_50_ reported for HO-AAVPA (192.4 μM)^13^. Furthermore, HO-AAVPA-PAMAM-G4 complex is more cytotoxic in 3T3 NIH cell lines than these compounds were tested separately which could be due to the better HO-AAVPA water solubility (Fig. 8). These mean that PAMAM-G4 could improve considerable the biological properties of HO-AAVPA as are reported for other small molecules^53^.

## Conclusion

Some drugs have poor solubility in water, as well as being unstable at certain pH conditions, some of them with anticancer properties, such as our HO-AAVPA compound. This led us to the evaluation of the interaction of HO-AAVPA with PAMAM-G4 dendrimer. The work presented here proposes a direct method to assess the quantitative structure-affinity relationship in a dendrimer-drug system by in silico studies (theoretically) confirmed by HPLC and by MS (experimentally). As expected, in basic and neutral medium, HO-AAVPA showed the highest affinity for PAMAM-G4, while in acidic medium, OH-AAVPA showed the weakest affinity. In addition, HO-AAVPA-PAMAM-G4 complex maintained its antiproliferative effects in the MCF-7 cell line and protected HO-AAVPA from any type of degradation (acid, basic, heat, light, and oxidative). These results allow pharmaceutical options to carry out HO-AAVPA in water conditions and protect the drugs from the biological environments including drug metabolism and drug deliver.

## Data Availability

The raw data is at*:* https://doi.org/10.5281/zenodo.7535687.
